# Is blue light exposure a cause of precocious puberty in male rats?

**DOI:** 10.3389/fendo.2023.1190445

**Published:** 2023-06-20

**Authors:** Aylin Kılınç Uğurlu, Aysun Bideci, Ayşe Mürşide Demirel, Gülnur Take Kaplanoğlu, Duygu Dayanır, Özlem Gülbahar, Tuba Saadet Deveci Bulut, Esra Döğer, Mahmut Orhun Çamurdan

**Affiliations:** ^1^ Ankara City Hospital, Pediatric Endocrinology Clinic, Ankara, Türkiye; ^2^ Department of Pediatric Endocrinology, Faculty of Medicine, Gazi University, Ankara, Türkiye; ^3^ Department of Basic Pharmaceutical Sciences Faculty of Pharmacy, Laboratory Animals Breeding and Experimental Research Center, Faculty of Pharmacy, Gazi University, Ankara, Türkiye; ^4^ Department of Histology and Embryology, Faculty of Medicine, Gazi University, Ankara, Türkiye; ^5^ Department of Medical Biochemistry, Faculty of Medicine, Gazi University, Ankara, Türkiye

**Keywords:** blue light (470 nm), precocious puberty, male rat, spermatogenesis, testis damage

## Abstract

**Purpose:**

Our study aimed to examine the effects of blue light exposure on prepubertal male rats’ puberty and testis tissue.

**Methods:**

Eighteen 21-day-old male Sprague Dawley rats were divided into three groups consisting of six rats in each group: Control Group (CG), Blue Light-6 hours (BL-6), and Blue Light-12 hours (BL-12). CG rats were maintained with 12/12-hour light-dark cycles. The rats of BL-6 and BL-12 were exposed to blue light (450-470nm/irradiance level 0.03uW/cm2) for 6 hours and 12 hours, respectively. Rats were exposed to blue light until the first signs of puberty. The ELISA method was used to analyze the serum levels of FSH, LH, testosterone, DHEA-S, leptin, ghrelin, melatonin, glutathione, glutathione peroxidase, and malondialdehyde. Testes were dissected for histomorphological examination.

**Results:**

The medians of the pubertal entry days of the CG, BL-6, and BL-12 were 38^th^, 30^th^, and 28^th^ days, respectively. (p:0.001) The FSH, LH, and testosterone concentrations of all groups were similar. The FSH concentration increased as the LH concentration increased (r: 0.82 p: 0.001). The serum LH concentration increased as serum testosterone, and DHEAS decreased, respectively (r: -0.561, p: 0.01) (r:-0.55 p:0.01). Testicular lengths and weights of the BL groups were smaller compared to CG (p: 0.03),(p: 0.04). GPx was higher for BL-6 and BL-12 than the CG (p:0.021, p:0.024). Testis tissue was compatible with the pubertal period in all groups. As the blue light exposure time increased, spermatogenesis was suppressed, and capillary dilatation and edema in the testis tissue increased.

**Conclusion:**

Our study is the first to show the effects of blue light exposure on male rats’ puberty process. And we showed that exposure to blue light and the duration of exposure lead to precocious puberty in male rats. The blue light exposure suppressed spermatogenesis, marked vasodilatation in the interstitial area of the testis, and disrupted the integrity of the basement membrane. These findings intensified with increasing exposure time.

## 1 Introduction

The primary source of blue light during the day is the sun. Blue light improve cognitive functions, mood, and alertness in the daytime. Blue light sources at night, such as fluorescent, LED, and television, have become prominent throughout the previous century. Though, during the last ten years, people of all ages have expanded the usage of touchscreen devices like tablets and smartphones ((1). These mobile touch devices emit high-energy, short-wavelength blue light. Because of the reduced eye-screen distance, blue light exposure is more substantial with these items. In recent years, the age of use of these devices in children has fallen rapidly (2). We know that the light-exposed at night has a suppressive effect on melatonin. In comparison to the impacts of other light wavelengths, the blue light had the most suppressive effect on melatonin. (3). And also the exposure to blue light at night is a stress factor and induces oxidative processes in tissues (4–6). Puberty is a phase of tremendous hormonal, behavioral, and physical changes which a child acquires reproductive function(7). The age of puberty is shifting, and there are rising worries about earlier puberty due to an increased risk of obesity, depression, and psychological issues (8). Genetic and environmental factors may impact the onset of puberty in animals, such as diet, chronic illnesses, geographic location, stressful situations, and pollution (9, 10). Moreover, one of these factors is light exposure, which has recently been intensively researched as a possible contributor to the diversity in puberty timing (11). However, the impact of environmental stressor blue light exposure on the prepubertal period and pubertal process are unclear. It has been reported that cases with precocious puberty from different continents have increased in the Covid 19 pandemic (12–17). It was thought that the cause of the increase in precocious puberty incidence could be associated, the limitation of movement, changes in eating habits, obesity, increased screen exposure, and stress (18). Various studies have revealed that obesity is not associated with this situation (19; 15 **;** 20; 21). While the screen time of children increased during this period, it was emphasized in some studies that the increase in screen time would be a factor in cases with precocious puberty(12; 14). It is challenging to elucidate the triggering mechanism that causes this with retrospective studies. The effect of blue light on puberty has become a more curious and concerned issue, especially in the Covid-19 pandemic.

Our previous study investigated the effects of blue light exposure and exposure time on puberty onset in female rats (22). The findings revealed that increased blue light exposure led to early puberty. Additionally, the study demonstrated a correlation between blue light exposure and decreased melatonin secretion, and highlighted the potential role of blue light-induced stress and its contribution to early puberty. Due to the higher frequency of organic causes in males and idiopathic causes in females in the etiology of precocious puberty, our study was initially conducted in female rats. We aimed to experimentally demonstrate the effect of exposure to blue light and the increase in exposure time on precocious puberty in male rats, to investigate the impact of hormones and oxidative stress that may cause it.

## 2 Materials and methods

### 2.1 Animals

Eighteen immature 21-day-old male Sprague Dawley rats weighing 35-50 g were procured from the Experimental Animal Center of Gazi University (Ankara, Turkey). The rats were housed in polysulfone cages (42.5 × 26.6 × 18.5 cm in size; 3 rats per cage) at 21-24°C and 40-45% humidity at the Laboratory Animals Breeding and Experimental Research Center of the Faculty of Pharmacy, Gazi University (Ankara, Turkey). The animals were fed a standard pellet diet and water *ad libitum* during research. All the animals were maintained by the Guide for the Care and Use of Laboratory Animals (23), and the experimental procedures were approved by the Experimental Animal Ethics Committee of Gazi University.

### 2.2 Light exposure protocol

**Figure 1:**
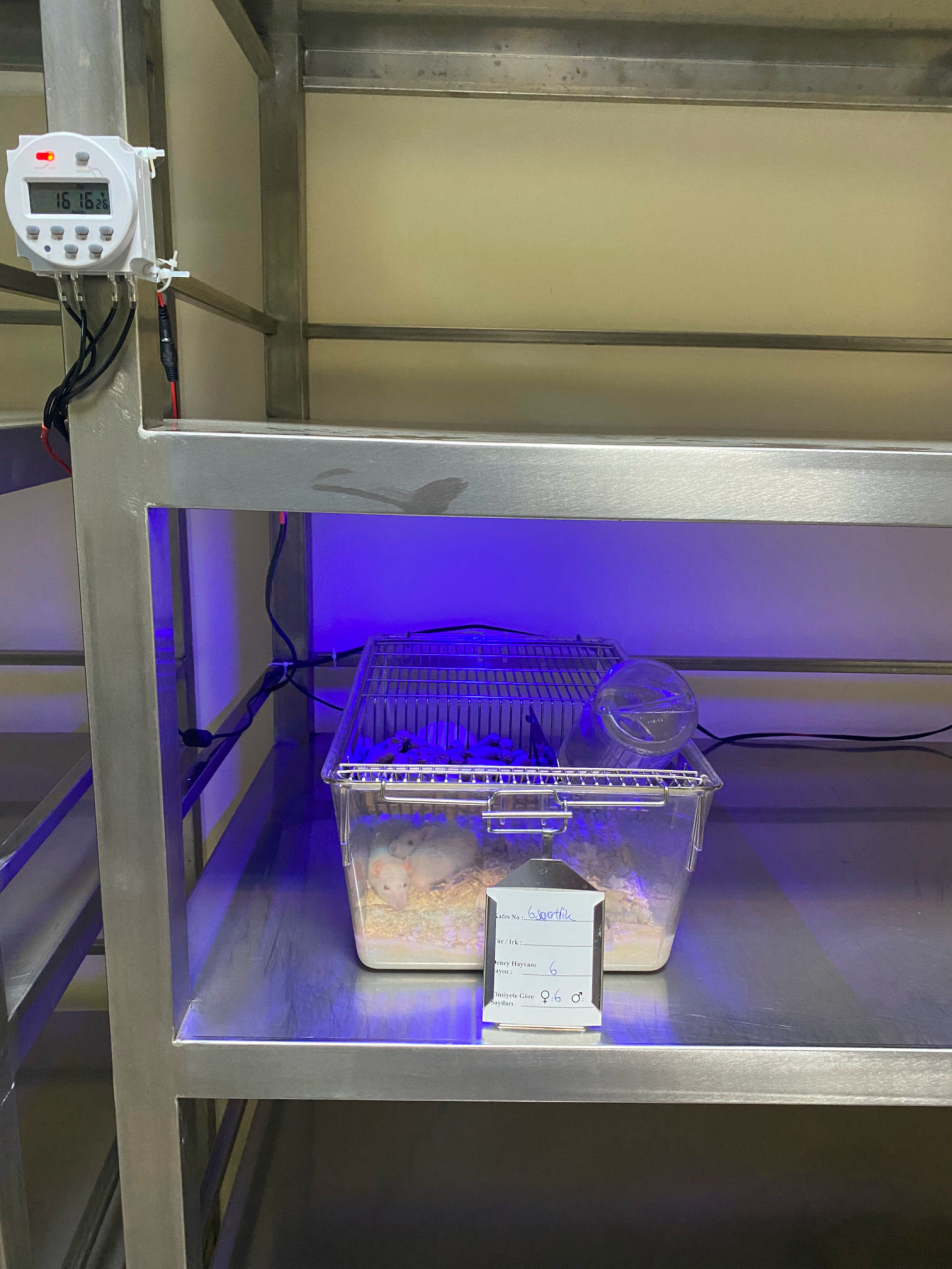
Experimental setup of room.

A blue LED strip (FSHI.1048.B020.6012, HI-LED, FLEX honor) providing blue light at a wavelength of 450-470 nm was placed approximately 20 cm above the center of each cage in the experimental groups (**Figure 1**).

In the experimental setup, the blue light source was used at an irradiance level that lowered rat melatonin concentrations but would not cause retinal damage (24–26). This was determined to be an irradiance level of 0.03 uW/cm^2^ at the rats’ eye level. The irradiance in the entire area of the cage where the rats were housed was measured with a spectroradiometer and adjusted to the same level.

### 2.3 Experimental design

The rats were randomly divided into three groups of six rats: the Control Group (CG), Blue Light-6 hours (BL-6), and Blue Light-12 hours (BL-12). CG rats were maintained under standard conditions with 12/12-hour light-dark cycles (light time 6:00 a.m.- 6:00 p.m.; dark time 6:00 a.m.-6:00 a.m.). The rats’ light/dark cycle condition in BL-6 were exposed to blue light (450-470 nm) for 6 hours (light time 6:00 a.m.- 6:00 p.m.; blue light time 6:00 p.m.-12:00 p.m.; dark time 12 p.m.-6:00 a.m.). The rats’ light/dark cycle condition in BL-12 were exposed to blue light (450-470 nm) for 12 hours (light time 6:00 p.m.-6:00 a.m.; blue light time 6:00 p.m.-06.00 p.m.).

The rats were weighed at the beginning and end of the experimental procedure, and the percentage weight gain was calculated with the formula of Weight gain (%)=(Last day–First day)/First day.

### 2.4 Termination of the experimental procedure

The first sign of puberty for male is preputial separation (27). The rats were examined daily, starting at 22 days of age, to detect preputial separation. Rats were exposed to blue light until the preputial separation.

After preputial separation, all the rats were sacrificed by taking blood from the heart at 8:00 p.m. according to determine the peak melatonin rhythm of rats (28) under general anesthesia (10 mg/kg xylazine hydrochloride and 50 mg/kg ketamine hydrochloride). After the anesthesia procedure, blood samples were obtained by the intracardiac puncture. The blood samples were centrifuged at 3000 rpm (906xg) for 15 minutes and the serum was separated. The serum samples were stored at -80°C until analysis. The height of the testes tissues was measured by a micrometer, and the testes tissues were dissected and weighted.

### 2.5 Determination of biochemical parameters

The collected blood was centrifuged at 3000 rpm for 10 minutes at +4°C and stored at -80°C. The serum concentration of the follicle-stimulating hormone (FSH), luteinizing hormone (LH), testosterone, Dehydroepiandrosterone sulfate (DHEA-S), leptin, ghrelin, melatonin, the antioxidant markers glutathione, and glutathione peroxidase (GPx), and the oxidative stress marker Malondialdehyde (MDA) were evaluated by enzyme-linked immunosorbent assay (ELISA; Rat kit; Bioassay Technology Laboratory, China).

### 2.6 Histopathological method

The right and left testis were measured and weighed after dissection, then the testes were fixed in Bouin’s fixative and embedded in paraffin blocks using standard procedures. Sections of 4-5micron thickness were taken from the prepared paraffin blocks and stained with hematoxylin and eosin. The samples were examined for histomorphological changes by light microscopy in the Leica DM4000 (Germany) computer-assisted imaging system, and images were obtained using the Leica-Qwin program.

### 2.7 Statistics

Statistical analysis was performed using IBM SPSS Statistics version 26 (IBM, Armonk, NY). The “Kruskal-Wallis” test was used when comparing the medians of three independent groups in the data that did not fit the normal distribution, and the “Mann-Whitney U” test were used when comparing the medians of two independent groups. While the investigating the associations between non-normally distributed and/ordinal variables, the correlation coefficients and their significance were calculated using the “Spearman test”. All data are given as mean ± SD Bonferroni correction was used in *post hoc* tests. Statistically, p<0.05 was considered significant.

A power analysis was performed using G-Power version 3.1.9.7 to determine the minimum sample size required to test the study hypothesis. Results indicated that a sample size of n=18 is required to achieve 80% power for detecting a large effect at a significance of α =0.05.

## 3 Results

At the beginning of the study, the mean weight of the male rat groups CG, BL-6 and BL-12 were 45,3 ± 2,9 g, 41,3 ± 3,2 g and 42,4 ± 6,6 g, respectively (p:0.20). The median time of preputial separation in male rats was 38 ^th,^ 30 ^th^, and 28 ^th^ days in CG, BL-6, and BL-12. The day of the beginning of puberty was statistically significantly earlier in BL-12 than in the CG (p:0.0001). The age of onset of puberty decreased as the duration of blue light exposure increased (r: -0.97, p: 0.001). The medians of weight gain (%) were 119.7 ± 18.5%, 125.2 ± 16,7%, 118.2 ± 30,7% in CG, BL-6, and BL-12 (p:0.84*).* Serum concentrations of leptin were lower in BL-12 compared to BL-6 *(p: 0.003)*. Ghrelin concentrations of groups are similar (p>0.05) (**Table 1**). There was no correlation between weight gain (%) and leptin (p>0.05), although serum leptin concentrations decreased as the onset of puberty progressed earlier (r:0.53, p:0.02).

**Table 1:** Puberty entry day, weight gain (%) and leptin, ghrelin concentrations of groups.

	Control Group	BL-6 Group	BL-12 Group	*p value*
*Puberty-on set(day)*	38± 0.9	30± 0.0	28.2 ± 0.4	**0.0001**
*Weight gain (%)*	119.7 ± 18.5	125.2 ± 16.7	118.2 ± 30.7	0.84
*Leptin (ng/mL)*	3.3 ± 0.3	3.6 ± 0.3	2.8 ± 0.3	*0.003
*Ghrelin (ng/L)*	2006 ± 401	1743 ± 107	1854 ± 287	0.37

Values represent mean ± SD.

^*^ BL-6 group vs. BL-12 group.

Serum concentrations of FSH, LH, testosterone, and DHEA-S were comparable between BL-groups and CG (p>0.05) (**Table 2**). The FSH concentration increased as the LH concentration increased (r: 0.82 p: 0.001). The serum LH concentration increased as serum testosterone, and DHEAS decreased, respectively (r: -0.56, p:0.01) (r: -0.55 p:0.01). Moreover, while the concentration of DHEAS declined, testosterone levels decreased. (r:0.53 p:0.02).

**Table 2:** Hormone serum concentrations of groups.

	Control Group	BL-6 Group	BL-12 Group	*p value*
*FSH (IU/mL)*	13.2 ± 9.2	9.3 ± 4.9	16.2 ± 2.7	0.09
*LH (IU/mL)*	70.1 ± 45.6	80.8 ± 8.4	100.7 ± 19.6	0.18
*Testosterone (nmol/L)*	263.8 ± 52.3	243.9 ± 30.9	222.8 ± 32.6	0.25
*DHEA-S (µmol/L)*	1.3 ± 0.2	1.2 ± 0.3	1.1 ± 0.2	0.19

Mean melatonin levels were 128.4 ± 23.4ng/L in CG, 144.1 ± 29 ng/L in BL-6, and 139.7 ± 14.3 ng/L in BL-12 (p>0.05). The mean ± SD of Glutathione levels were 166.8 ± 28.8mg/L, 173.1 ± 27.3 mg/L, 183.1 ± 30,3 mg/L, in the CG, BL-6, and BL-12; MDA levels were 1± 0.3 nmol/mL, 1.1 ± 0.2 nmol/mL, 1.1± 0.2 nmol/mL,1.2 ± 0.1 nmol/mL in the CG, BL-6, and BL-12, respectively(p>0.05). The mean ± SD of GPx levels were 85.9 ± 11.4 U/mL, 115.9 ± 22.8 U/mL, 188 ± 3.2 U/mL in the CG, BL-6, and BL-12. It was found to be statistically significantly higher for BL-6 and BL-12 compared to the CG (p=0.02, p=0.02). (**Table 3**).

**Table 3:** Melatonin, antioxidant, and oxidative markers’ concentrations of groups.

	Control Group	BL 6 Group	BL 12 Group	*p value*
*Melatonin (ng/mL)*	128.4 ± 23.4	144.1 ± 29	139.7 ± 14.3	0.52
*Glutatyon (mg/L)*	166.8 ± 28.8	173.1 ± 27.3	183.1 ± 30.3	0.71
*GPx (U/mL)*	85.9 ± 11.4	115.9 ± 22.8	108.9 ± 3.2	*0.008
*MDA (nmol/mL)*	1 ± 0.3	1.1 ± 0.2	1.1 ± 0.2	0.72

GPx, Glutathione peroxidase; MDA, Malondialdehyde.

Values represent mean ± SD.

^*^ Control group vs. BL-6 group and BL-12 group.

Testicular lengths of the CG were longer than the BL groups (p=0.03). Although weight of testes was higher in the CG than the BL groups (p=0.04) (**Table 4**).

**Table 4:** Testicular weight and length of groups.

	Control Group	BL 6 Group	BL 12 Group	*p value*
*Right testicular weight (mg)*	0.6 ± 0.1	0.5 ± 0.1	0.5 ± 0.1	*0.02
*Left testicular weight (mg)*	0.6 ± 0.1	0.5 ± 0.1	0.5 ± 0.1	*0.02
*Right testicular length (mm)*	1.5 ± 0.1	1.3 ± 0.1	1.3 ± 0.1	0.06
*Left testicular length (mm)*	1.5 ± 0.1	1.3 ± 0.1	1.3 ± 0.0	*0.03

Values represent mean ± SD.

^*^ Control group vs. BL-6 group and BL-12 group.

**Figure 2:**
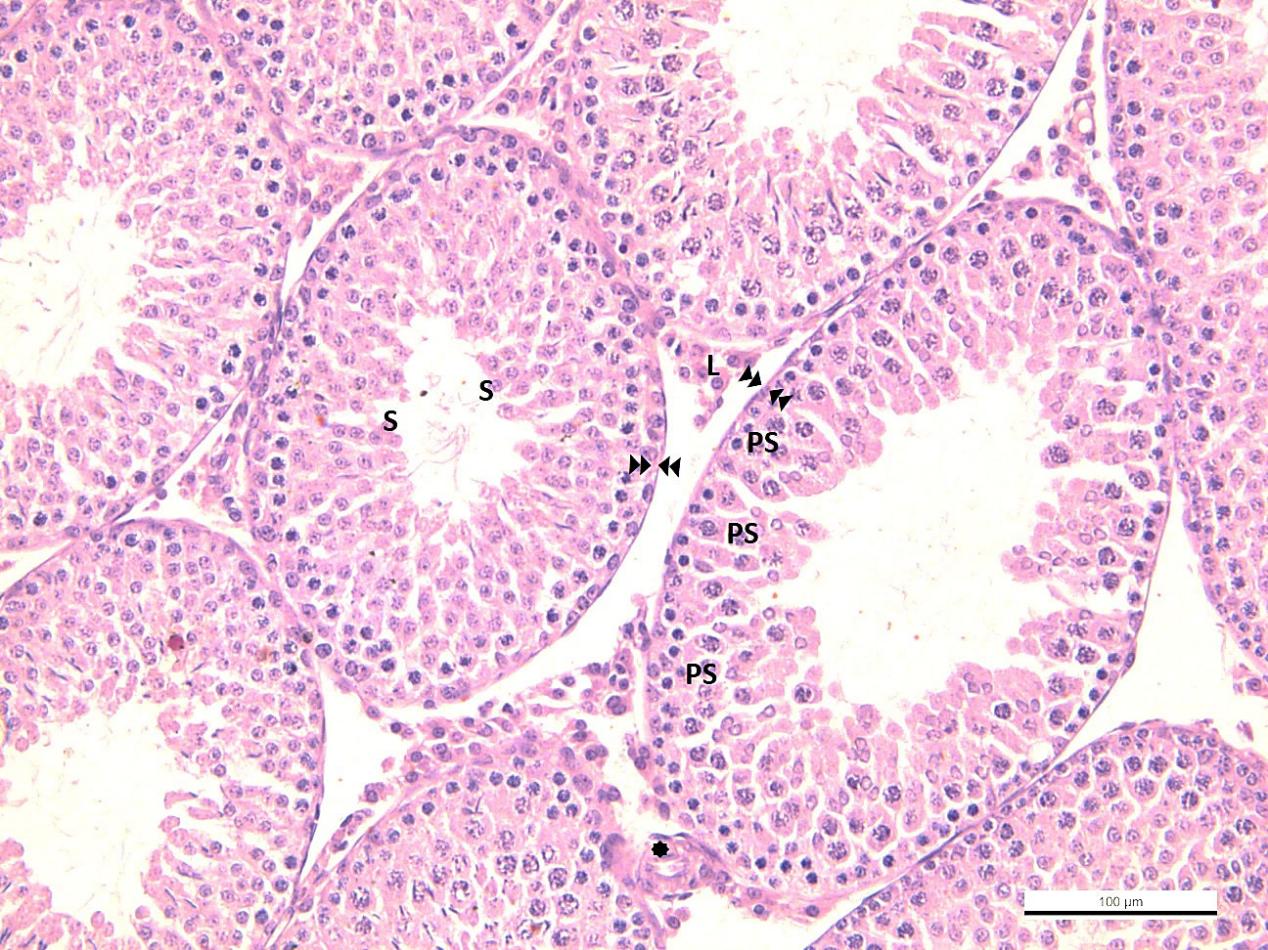
**-CG** 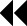: seminiferous tubule basement membrane in normal configuration 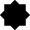: blood vessel in normal configuration Sp: spermatogonium, PS: primary spermatocyte, S: secondary spermatocyte and spermatid, L: Leydig cell.

In the testicular tissue of the control group, spermatogonium located in the apical part of the basement membrane of the seminiferous tubule, primary spermatocytes settled in the upper compartment, and secondary spermatocytes located close to the lumen were observed with typical histological structures. In one group, thick seminiferous epithelium and spermiums were observed in the lumen, indicating that the spermatogenic cycle was completed. In another group, spermatogenesis is not fully completed; A thin wall distinguished it, and a wide lumen and absence of spermiums distinguished it. Leydig cells with eosinophilic cytoplasm and vascular structures were evident in the interstitial area (**Figure 2**).

**Figure 3:**
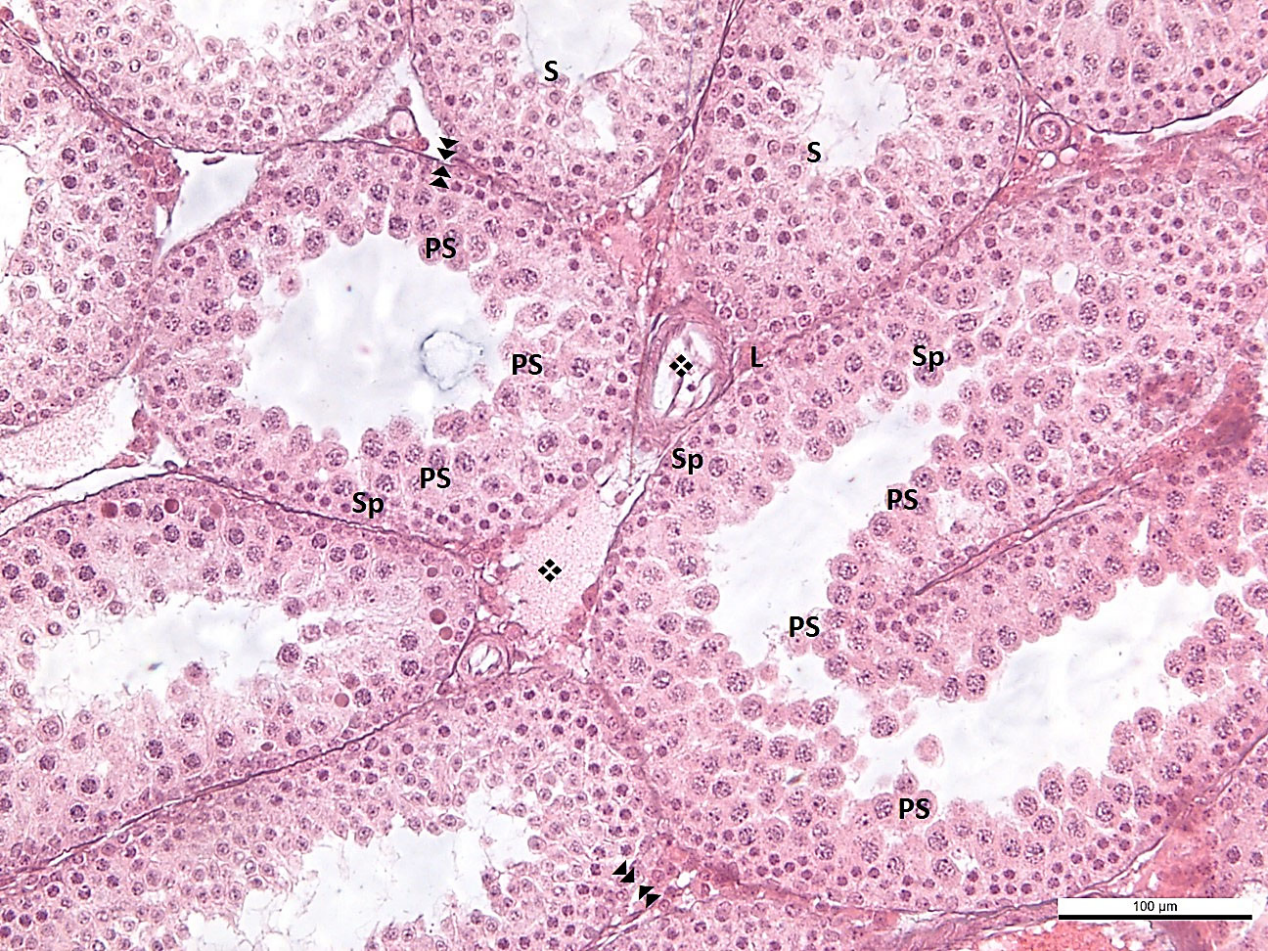
**-BL6** 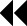: effacement, thickening and corrugation of the basement membrane of the seminiferous tubule. 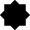: blood vessel in normal configuration ❖: vasodilatation Sp: spermatogonium, PS: primary spermatocyte, S: secondary spermatocyte and spermatid, L: Leydig cell.

In the BL-6 group, the length of the spermatogenic epithelium was short, and the cell content consisted of spermatogonium and primary spermatocytes in the seminiferous tubules. This finding is considered an indication of incomplete spermatogenesis in most tubules. In addition, in the tubules where spermatogenesis was not yet completed, irregularity in the basement membrane structure, erasure in some areas, and thickening and corrugation in some fields were evident. In several tubules, spermatogenesis was completed with wall thickness and spermatids observed in the lumen. Extensive edema and vascular dilatations were remarkable in the interstitial area. Leydig cells were observed compressed in the periphery of dilated vessels (**Figure 3**).

**Figure 4:**
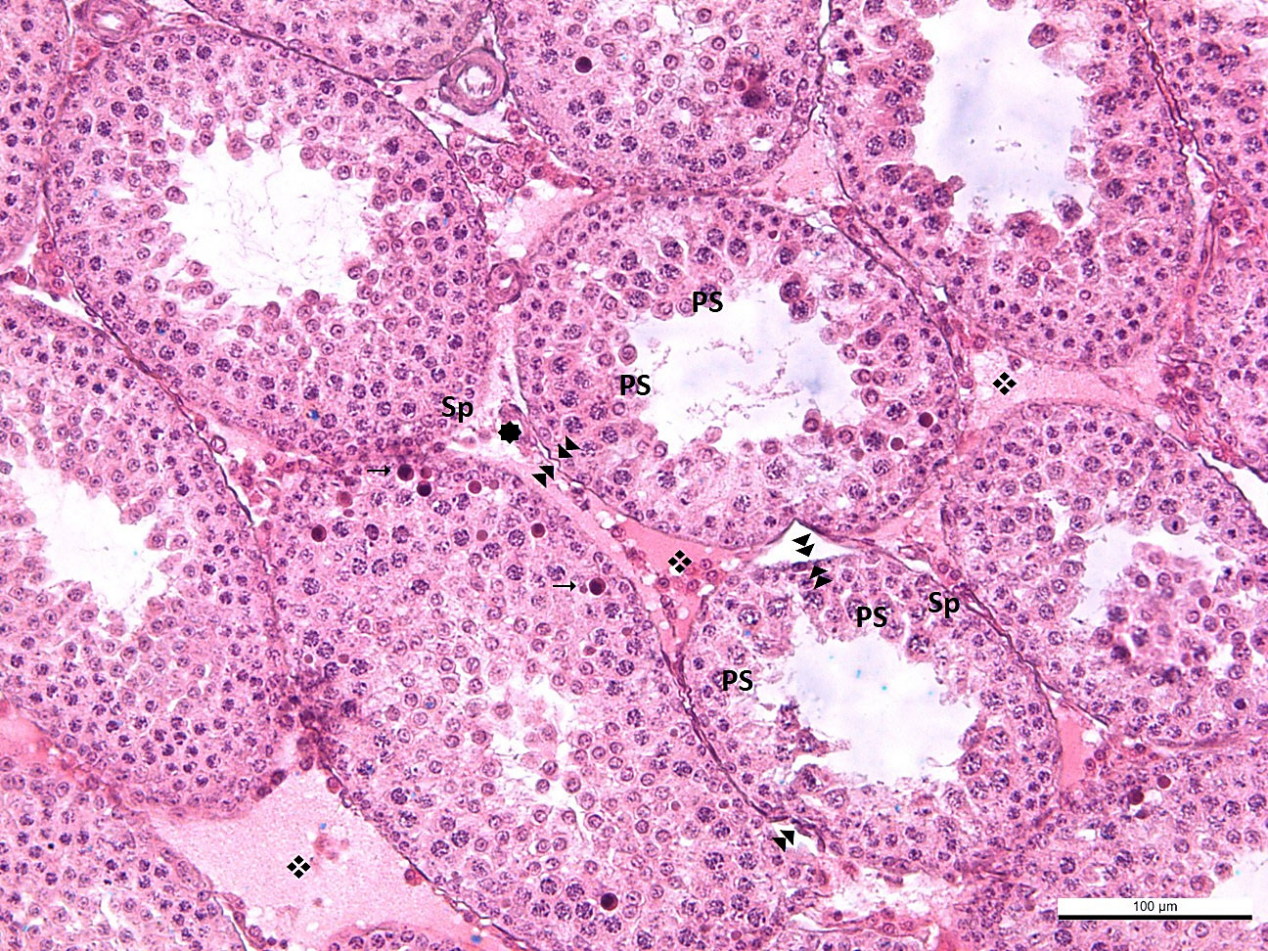
**-BL12** 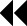: effacement, thickening and corrugation of the basement membrane of the seminiferous tubule. 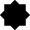: blood vessel in normal configuration ❖: vasodilatation →: leukocytes in the seminiferous tubule wall Sp: spermatogonium, PS: primary spermatocyte, S: secondary spermatocyte and spermatid, L: Leydig cell.

All findings observed in the BL6 group were also detected in the BL-12 group. In the BL12 group, edema was observed in some seminiferous tubule walls, and some of their epithelium was shed into the lumen. Separation of the basement membrane was evident in some tubules. The number of tubules in which spermatogenesis was completed was low. In this group, degenerative appearances in the form of corrugations, especially effacements in the basement membrane, were quite common. In the interstitial area, capillary dilatation was very common, and congestion was present in this group. Leucocytes were prominent among the seminiferous epithelial cells. This finding indicates that the permeability of the basement membrane with impaired integrity was adversely affected, and the structure of the blood-testis barrier was impaired. Normal-appearing Leydig cells were detected both alone and in clusters (**Figure 4**). The structure of Sertoli cells was typical in all groups. There was a significant decrease in the number of seminiferous tubules completing spermatogenesis with an increase in blue light exposure.

Thus, spermatogenesis, an indicator of puberty, was detected in all groups. The results showed that blue light exposure inhibited spermatogenesis, produced significant vasodilation in the interstitial region of the testis, and compromised the basement membrane’s integrity. It was concluded that these findings were exacerbated with increasing exposure time.

## 4 Discussion

In our study, exposure to blue light accelerated the onset of puberty in male rats. When the length of exposure to blue light increased, the onset of puberty occurred earlier. To best of our knowledge, this study is the first to demonstrate the effects of blue light exposure on male rats’ puberty onset.

One of the cornerstones of human evolution, the mechanisms regulating the onset of puberty have not yet been fully understood. However, a significant collection of data has given insight into various essential components of the process. Various factors such as genetics, epigenetics, and lifestyle play a role in the onset and completion of puberty (29). Stress and energy balance also have significant roles in this process. We know that throughout this phase of life, many factors interact dynamically to guide a more comprehensive understanding of the mechanisms regulating the onset of puberty. While idiopathic etiology is the most common cause in girls, central nervous system (CNS) tumors or injuries are more frequent in boys. It is thought to be since girls are more affected by environmental factors. In the publications on precocious puberty during the pandemic, the increased incidence has been reported primarily on girls. However, publications are reporting an increase in the incidence of male cases. (15, 30). Researchers have attempted to attribute the increase in pandemic cases to the change in environmental conditions. Our study aims to provide valuable insights by experimentally demonstrating the impact of blue light on the increased incidence of precocious puberty cases during the pandemic period, utilizing rats as a preferred animal model for preclinical *in vivo* research due to their ability to mimic human physiology (31, 32). At this point in our research, we found evidence that blue light triggers central puberty. The study demonstrated that both control and BL groups exhibited a central onset of puberty, as confirmed by physical examination, tissue samples, similar gonadotropin and testosterone concentrations in the BL groups. The positive correlation between the increase in LH and FSH supported the central onset of puberty. In addition, completed *spermatogenesis* in the seminiferous tubules of BL groups validated puberty. In our previous study (22), we found that exposure to blue light in prepubertal female rats caused an earlier onset of puberty. The results of these studies support that prepubertal blue light exposure causes precocious puberty in both female and male rats.

Blue light has been in the life of humans since the use of color screens in daily life. With the increase in the use of mobile phones and tablets in childhood in the last decade, blue light exposure has increased even more (1, 2, 33). It is the first study to show a direct relationship between blue light and puberty in males. It has been mentioned in previous publications that blue light suppresses leptin and causes an increase in appetite, and as a result, it causes an increase in adipose tissue (34, 35). Although leptin increase is mentioned as one of the reasons that initiate puberty, its active role in the onset of puberty is still unclear.

In previous studies, leptin stimulates LH secretion, but it exerts this effect through neuropeptides such as neuropeptide Y (NPY), agouti-regulated peptide (AgRP), proopiomelanocortin (POMC), and gamma-aminobutyric acid (GABA) found in GnRH neurons (36). GnRH cells do not have leptin receptors (37). It provides its effects on the reproductive system through neuropeptides. And also, there are publications showing that it is not due to its metabolic effect (38).

In addition, many studies demonstrate that leptin regulates the reproductive system *via* regulating the Kiss1 gene’s synthesis of kisspeptins (37).

The lower leptin level in the BL-12 group compared to the BL-6 group may be due to the suppressive effect of blue light on leptin. In our study, the earlier onset of puberty was observed as the levels of leptin decreased. Some studies have shown that leptin levels increase in the prepubertal period but decrease to basal levels at the beginning of puberty (39, 40). The decrease in leptin levels at the onset of puberty may be associated with this situation.

Rapid weight gain causes a decrease in ghrelin concentrations in contrast to leptin (41). Ghrelin suppresses pulsatile GnRH secretion; therefore, it can play essential roles in pubertal onset (42, 43). The ghrelin concentrations in the BL groups were lower than CG, but it was not statistically significant. Chen et al. found that the increase in the incidence of precocious puberty in Shanghai during the pandemic may be associated with decreased concentrations of MKRN3 and ghrelin. In this study, it was found similar in terms of BMI in the pandemic and pre-pandemic groups (44).

We found no evidence that weight gain had an impact on precocious puberty in male rats. Studies have also noted the association of elevated BMI with earlier puberty in girls, although the relationship between BMI and the onset of puberty in boys is less consistent (45). A previous study we conducted in prepubertal female rats determined that weight gain had no effect on precocious puberty (22). In addition, the fact that external administration of leptin initiates the onset of puberty without affecting body weight also supports that the effect of leptin on the onset of puberty is independent of weight and energy storage (46).

Blue light exposure reduces melatonin release (47). Melatonin secretion peaks in the evening in both diurnal and nocturnal animals (26). A prior study found that plasma melatonin decreased with increasing age at the start of puberty. The decline in serum melatonin may help activate the hypothalamic pulsatile GnRH, which induces the reproductive axis and promotes the onset of puberty (48).

There was no difference between the melatonin concentrations of the groups. In our previous study investigating the effect of blue light on prepubertal female rats, a precocious puberty was associated with a decrease in melatonin with exposure to blue light (22). In our study, we sacrificed rats at the time when melatonin levels started to rise (28). In melatonin research, collecting control serum samples at regular intervals is more appropriate to maintain accurate results. However, due to the tiny size of the rats, we could not obtain repetitive serum samples. We know that blue light has the most suppressive wavelength on melatonin(3). However, our study did not detect any difference in melatonin levels at the time of sacrificing rats. One of the limitations of our investigation was that we could not collect samples at regular intervals.

We found that a size and weight of testicular tissue of the BL groups smaller than the control group. In cases of blue light, it can be explained by the suppression of spermatogenesis and the inability to grow the thickness of the seminiferous tubules during the evolution of the pubertal process of the tissue. In addition, with the increase in blue light exposure, testosterone, and DHEAS levels decreased, while LH increased physiologically. DHEAS is a precursor of testosterone; hence the fact that testosterone levels declined with DHEAS levels.

Spermatogenesis is mainly influenced by LH and FSH secreted by the anterior pituitary gland, and testosterone secreted from Leydig cells. Testosterone is required to grow and divide testicular germinal cells, the first step for sperm formation (49). Leydig cells are located outside the seminiferous tubules and contain LH receptors. Transcriptome analysis of testicular tissue from LH receptor (LHR) knockout (LuRKO) mice treated with LH receptor KO (LuRKO) and testosterone showed that most of the defects in testicular gene expression in the absence of LH actions were largely corrected by testosterone replacement (50, 51).[53] For a long time, it was believed that FSH is a more critical factor for spermatogenesis than LH. However, findings from LuRKO mice revealed the importance of LH for spermatogenesis. However, the dominant gonadotropin information for spermatogenesis has begun to shift from FSH to LH (52, 53). Testicular germ cell development, crucial for spermatogenesis, is influenced by the secretion of LH and FSH from the anterior pituitary gland, as well as testosterone from Leydig cells, which is necessary for the growth and division of germinal cells. Elevated exposure to blue light may potentially suppress spermatogenesis by diminishing intratesticular testosterone production. We observed a significant decrease in the number of seminiferous tubules completing spermatogenesis with an increase in blue light duration. In this process, we can assume that the decrease in testosterone, LH increase, and the histological absence of differentiation in Leydig cells prevent the release of blue light to testosterone at the cellular.

In our study, dysmorphological findings such as edema and increased vascularity in the interstitial area of the testicular tissue, deterioration in the integrity of the seminiferous tubule basal membrane, effacement, and corrugation were observed in rats with exposure to blue light. It has been observed that blue light causes damage to the testicles in studies in which 48 to 72 hours of phototherapy (blue light) were applied to newborn rats (4, 54). Krishna et al. (54) showed that normal phototherapy exposure to 48-hour-old Wistar rat pups caused degenerative ultrastructural changes in the seminiferous tubules. These changes were most noticeable at PND 70 and 100, but by PND 130, these cells were almost completely recovered. These effects may be due to prolonged light triggering oxidative processes in the tissue. However, the MDA value was similar between the groups in our study. The level of GPx, an antioxidative in response to the oxidative process, was found to be higher in BL groups than in CG. In order to understand the long-term effects of blue light on testis damage and fertility, rats should be followed up to advanced ages.

In conclusion, blue light exposure caused precocious puberty in male rats. It was concluded that blue light application caused marked vasodilatation in the interstitial area of the testis, disrupted the integrity of the basement membrane, and these findings intensified with increasing exposure time.

In addition, showing the effect of blue light on GnRH, neuropeptides, kisspeptin, and kisspeptin receptors, which are involved in the onset of puberty, will be more guiding in illuminating this issue. Further studies are needed to confirm this hypothesis and clarify the underlying causative mechanism.

## Data availability statement

The raw data supporting the conclusions of this article will be made available by the authors, without undue reservation.

## Ethics statement

The animal study was reviewed and approved by Experimental Animal Ethics Committee of Gazi University.

## Author contributions

AU contributed to the conceptualization of the study, design of the experiments, data collection, data analysis, and writing of the manuscript. AB contributed to the conceptualization of the study, design of the experiments, data analysis, and critical review of the manuscript. AD contributed to the design and execution of laboratory experiments, as well as the interpretation of the study results. GK contributed to the preparation and analysis of histological samples, as well as the interpretation of the study results. DD contributed to the preparation and analysis of histological samples, as well as the interpretation of the study results. ÖG contributed to the investigation and analysis of biochemical data, as well as the interpretation of the study results. TB contributed to the investigation and analysis of biochemical data. ED contributed to the interpretation of the study results. MÇ contributed to the critical review of the manuscript. All authors listed have made a substantial, direct, and intellectual contribution to the work and approved it for publication. 
